# Supplementary material for: Complexome profiling of the Chlamydomonas psb28 mutant reveals TEF5 as an early PSII assembly factor
Source: Plant Cell. 2025 Mar 17;37(6):koaf055. doi: 10.1093/plcell/koaf055 (PMC12164590; doi:10.1093/plcell/koaf055)
Supplement: koaf055_Supplementary_Data [file koaf055_supplementary_data.zip › psb28_interactive.zip!/plots/intensity/qualityPlots/peptides/PSAN_Cre02.g082500.t1.1.html]

### Cre02.g082500.t1.1

Trivial name: PSAN  
SeqCoverage [median]: 20.90  
Mass: 14.80 kDa  
  
 

| Sequence | MissedCleavage | Mass [Da] | wtIntensity | muIntensity | Score |
| --- | --- | --- | --- | --- | --- |
| LATSYANLAR | 0 | 1078.58 | 451842.79 | 226207.61 | 243.38 |
| RLATSYANLAR | 1 | 1234.68 | 8412.82 | 4774.39 | 169.58 |
| SAANKALNDKKR | 3 | 1314.74 | 0.00 | 1174.12 | 113.61 |
|  |  |  | 460255.6 | 232156.1 |  |
